# *Pseudomonas alcaliphila* NEWG-2 as biosorbent agent for methylene blue dye: optimization, equilibrium isotherms, and kinetic processes

**DOI:** 10.1038/s41598-023-30462-w

**Published:** 2023-03-05

**Authors:** Abeer A. Ghoniem, Zeiad Moussa, Asma Massad Alenzi, Amenah S. Alotaibi, Hala Fakhry, Ayman Y. El-Khateeb, WesamEldin I. A. Saber, Ashraf Elsayed

**Affiliations:** 1 Microbial Activity Unit, Department of Microbiology, Soils, Water and Environment Research Institute, Agricultural Research Center, Giza, 12619 Egypt; 2 Genomic and Biotechnology Unit, Department of Biology, Faculty of Science, University of Tabuk, Tabuk, Saudi Arabia; 3 Polymer Materials Research Department, Advanced Technology and New Material Institute, City of Scientific Research and Technological Applications (SRTA-City), Alexandria, 21934 Egypt; 4 National Institute of Oceanography and Fisheries (NIOF), Cairo, Egypt; 5 Agricultural Chemistry Department, Faculty of Agriculture, Mansoura University, Mansoura, Egypt; 6 Botany Department, Faculty of Science, Mansoura University, Mansoura, Egypt

**Keywords:** Microbiology, Applied microbiology, Bacteria, Bacteriology, Environmental microbiology

## Abstract

In comparison to physicochemical and chemical methods, microbial dye biosorption is regarded as an eco-effective and economically viable alternative and is a widely applied method due to its high efficiency and compatibility with the environment. Therefore, the idea of this study is to clarify to what extent the viable cells and the dry biomass of *Pseudomonas alcaliphila* NEWG-2 can improve the biosorption of methylene blue (MB) from a synthetic wastewater sample. The array of Taguchi paradigm has been conducted to ascertain five variables affecting the biosorption of MB by broth forms of *P. alcaliphila* NEWG. The data of MB biosorption were familiar to the predicted ones, indicating the precision of the Taguchi model’s prediction. The maximum biosorption of MB (87.14%) was achieved at pH 8, after 60 h, in a medium containing 15 mg/ml MB, 2.5% glucose, and 2% peptone, with sorting the highest signal-to-noise ratio (38.80). FTIR spectra detected various functional groups (primary alcohol, α, β-unsaturated ester, symmetric NH_2_ bending, and strong C–O stretching) on the bacterial cell wall that participated in the biosorption of MB. Furthermore, the spectacular MB biosorption ability was validated by equilibrium isotherms and kinetic studies (the dry biomass form), which were derived from the Langmuir model (q_max_ = 68.827 mg/g). The equilibrium time was achieved in about 60 min, with 70.5% of MB removal. The biosorption kinetic profile might be adequately represented by pseudo-second order and Elovich models. The changes in the bacterial cells before and after the biosorption of MB were characterized using a scanning electron microscope. As realized from the aforementioned data, the bacterium is a talented, effective, eco-friendly, and low-cost bio-sorbent for the decolorization and remedy of an industrial effluent containing MB from an aqueous environment. The current outcomes in the biosorption of MB molecules promote the use of the bacterial strain as viable cells and/or dry biomass in ecosystem restoration, environmental cleanup, and bioremediation studies.

## Introduction

Dye-polluted water is a major problem, and it is not informal to imagine red, blue, or brown colors among the parameters of water quality judgment. The environmental pollution caused by dyes results in aesthetic problems and severe public health concerns, tallying to several serious environmental issues, due to their residence in nature, and being deprived of biodegradability features. There are approximately more than 10.000 different commercially available dyes, with annual productivity of 7 × 10^5^ tons worldwide^1^. The pollution with dye is highly noticeable, even in low concentrations ˂ 1 ppm, which is detrimental, carcinogenic, and harmful to aquatic life and the food chain. Moreover, the dyes are usually stable to light, oxidizing agents, and tolerant to aerobic deprivation, with worries about degradability^2^. Recently, dyes, on the other hand, have an exciting comprehensive genuine concern, owing to their toxic effect on the ecosystem. Methylene blue (MB), is one of these dyes, which acts as a cationic dye, with exhaustion in the dyestuff of the textile industry, wood, and silk. It is also a medicament for methemoglobinemia and psoriasis^3^. MB assimilation causes respiratory issues, skin hurt, nausea, mental confusion, mutagenesis, jaundice, and teratogenesis^4^.

In other words, the removal of dyes within permissible limits has been considered a successful achievement. The customary strategies of dyes removal from the ecosystem embrace microbial systems^5,6^, membrane severance^7^, phytocure^8^, and sono-fenton pattern^9^, all these procedures could be viewed according to biological and financial principles. The adsorption process would be cracked since the most relevant system exhibited cost-effectiveness, versatility, operation flexibility, and reversibility. Various adsorbents, e.g. nanoparticles^9^, agronomic residues^10^, industrial effluent materials^11^, coagulation, electrochemical adsorption, photo-oxidizing, and triggered carbon had been investigated^12^. Due to its high surface area and porous structure, enthused carbon has been certified as an effective sorbent for water purification^13^.

However, its request is cramped due to its grand cost of production and regaining. Further, except for costly operations, generally, these techniques create secondary problems during the creation of dye-bearing sludge. The process is ineffective when the level of dyes exceeds the dosage over the capacity of the sorbent. Consequently, the occupation of low-priced materials as particular adsorbents for the recapture of dyes from wastewater has been highlighted. Whereby, the efforts of investigators have urgently contributed to finding out alternative adsorbents, with feasible, accessible, economical, and environmentally friendly.

The biosorption process is an incident that signifies to passive absorption of molecules such as metal ions and dyes by microbial cells^14^. The microbial community e.g., algae, bacteria, and fungi had been defined for such biosorption processes, in which they have merits of efficiency and outstanding reusability, as well as, potentiality towards dyes and metal ions^15,16^.

Several complex xenobiotic chemicals (dyes) generated by humans are reduced to simpler inorganic components or even mineralized into carbon dioxide and water by bacterial or fungal remediation. Such a natural phenomenon offers numerous benefits over physical and chemical processes, including eco-friendliness, financial effectiveness, and a reduction in the production of sludge or incompletely degraded byproducts, which add to the load on the environment^17^. The bioremediation relies on the use of plants, microbes, and/or their enzymes. In this context, diverse microbial forms (living, dry biomass) under various environmental setups have been combined or sequentially documented^18^.

Further, other studies found that the most promising microorganisms for biosorption and colorization of dyes showed to be endogenous organisms^19,20^. However, the biosorption of many dyes admitting exertion, which the bacteria grow fairly on dyes as a sole source of energy and carbon, the optimal co-substrate such as sugars are necessary for boosting the activity of bacteria against dyes breakage^21,22^. Zhang et al.^23^ found out that the fructose sugar was boosting for sorption of azo dye by *P*. *aeruginosa* DDMZ1-2. Interestingly, Kishor et al.^24^ demonstrated a significant removal of MB by *Bacillus albus* isolated from a textile sludge sample. Moreover, Eslami et al.^25^ reported that *Pseudomonas aeruginosa* removed about 82.25% of MB (50 mg/L) during 24 h of incubation. Furthermore, agro-industrial waste was used as an economical and nutritive to recruit a consortium of *Staphylococcus xylosus*, *Raoultella planticola*, and *Rhodotorula* sp. in the biodegradation of MB^18^.

A separate study examined how effective Pseudomonas aeruginosa was in biosorption and decolorization of Brown706 dye under varying physicochemical conditions^26^. Likewise, the efficiency of encapsulated *P*. *aeruginosa* in removing MB has been conducted^27^. The bio hydrolysis of MB by *Alcaligenes faecalis* had been conducted during batch and continuous processes using packing media^28^. The biosorption of MB dye has been investigated by the alga, *Enteromorpha prolifera*^29^. As well as, the efficiency of *Sargassum muticum* in biosorption of MB dye was reported^30^.

The classical approach of one factor at a time (OFAT) is a traditional design of experiments technique that studies the impact of each independent variable on the response variable by changing only one factor at a time. The Taguchi methods, on the other hand, are based on the philosophy of robust design and use a systematic design of experiments approach to study the effects of multiple factors simultaneously, considering the interactions between factors and identifying the optimal levels for the best response. The OFAT approach is limited and time-consuming, while the Taguchi methods provide a more comprehensive understanding of the process or system being studied^18^. The fractional factorial statistical Taguchi design is a robust and multi-optimization procedure. The design systematically rates experimentation and the data to gauge the optimum conditions of functioning, and quality. The design can realize the best-operating conditions of several variables with the lowest experimentation^31,32^. The Taguchi orthogonal arrays have been developed from the factorial designs and Latin squares (L), using assorted sets of variables^32,33^. In the matrix of the orthogonal array, each pair of factors appear equally in every pair of columns^33^.

The orthogonal array output is optimized based on the maximization of the signal-to-noise (*S*/*N*) ratio as an alternative to the response to minimize the experimental variability^34^. The signal is the experimental response value, while noise is the standard deviation. Therefore, the operating conditions that minimize the process variability (standard deviation) are accompanied by increasing the *S*/*N* ratio, where the S/*N* ratio detects the variance between the predictable (desired) value and the experimental response. Therefore, the S/N ratio directs the response to the optimum circumstances of control (signal) variables, consequently, ignored the deviations occured by the uncontainable (noise) factors^31,35^.

To the best of the authors' knowledge, no study has addressed the usability of *P. alcaliphila* NEWG-2 as a biosorbent material for the removal of MB dye. Herein, in this study, the ability of viable cells and dry biomass of *P. alcaliphia* forms as biosorbent of MB from an aqueous solution was investigated. The effectiveness of the viable- and dry-biomass-based *P. alcaliphia* strains were assessed using a factorial statistical Taguchi design, as well as equilibrium and kinetic studies, respectively. Furthermore, the biosorption behavior of the prepared bio-sorbent was confirmed by FTIR spectra and SEM images.

## Materials and methods

### Preparation of MB stock solution

The cationic dye MB was used as a contaminant dye model in this study. The process of creating a solution containing MB usually involves dissolving a defined amount of MB powder in doubly distilled water. A 500 ppm-concentrated stock dye solution was set in distilled water. The working MB concentrations (5–200 ppm) were prepared from the stock solution through the dilution process.

### Bacterium and medium

*Pseudomonas alcaliphila* NEWG-2 used in the subject research was formerly identified with GenBank accession number; MN025267^36^. The medium components were prepared at various levels (Table 1), followed by sterilization (121 °C, 15 min). A 0.22-µm membrane filter was used to sterilize a standard glucose solution before being inserted into the medium.

**Table 1:** Taguchi’s L_25_ (5^5^) orthogonal array, and S/N ratio for optimization of MB biosorption, using *P. alcaliphila* NEWG-2.

Run	Tested factors					MB removal (%)		S/N ratio
	pH	Incubation time (h)	Initial MB (mg/ml)	Glucose (%)	Peptone (%)	Actual	Predicted	
L1	5.5	24	10	0.5	1.0	56.68 ± 1.07	56.81	35.07
L2	5.5	36	15	1.0	1.5	61.58 ± 1.09	61.86	35.79
L3	5.5	48	20	1.5	2.0	64.70 ± 1.05	64.94	36.22
L4	5.5	60	25	2.0	2.5	74.14 ± 0.57	73.86	37.40
L5	5.5	72	30	2.5	3.0	71.87 ± 0.53	71.50	37.13
L6	6.5	24	15	1.5	2.5	66.68 ± 0.98	66.31	36.48
L7	6.5	36	20	2.0	3.0	68.47 ± 1.09	68.60	36.71
L8	6.5	48	25	2.5	1.0	75.59 ± 0.83	75.87	37.57
L9	6.5	60	30	0.5	1.5	70.18 ± 0.80	70.43	36.92
L10	6.5	72	10	1.0	2.0	67.70 ± 1.24	67.41	36.61
L11	7.5	24	20	2.5	1.5	72.84 ± 0.90	72.56	37.25
L12	7.5	36	25	0.5	2.0	74.58 ± 0.84	74.21	37.45
L13	7.5	48	30	1.0	2.5	77.20 ± 0.91	77.33	37.75
L14	7.5	60	10	1.5	3.0	75.47 ± 1.11	75.75	37.56
L15	7.5	72	15	2.0	1.0	76.18 ± 0.92	76.43	37.64
L16	8.5	24	25	1.0	3.0	80.25 ± 0.77	80.50	38.09
L17	8.5	36	30	1.5	1.0	83.16 ± 0.86	82.88	38.40
L18	8.5	48	10	2.0	1.5	80.84 ± 0.98	80.47	38.15
L19	8.5	60	15	2.5	2.0	87.14 ± 1.13	87.27	38.80
L20	8.5	72	20	0.5	2.5	81.79 ± 1.11	82.07	38.25
L21	9.5	24	30	2.0	2.0	80.10 ± 0.77	80.38	38.07
L22	9.5	36	10	2.5	2.5	83.40 ± 1.06	83.64	38.42
L23	9.5	48	15	0.5	3.0	79.50 ± 1.06	79.22	38.01
L24	9.5	60	20	1.0	1.0	83.80 ± 0.99	83.43	38.46
L25	9.5	72	25	1.5	1.5	85.80 ± 1.11	85.93	38.67

A fresh inoculum was prepared from a 48-h-old culture grown on the previous broth medium (at the middle concentration) under shaking (100 rpm) and at 28 ± 1 °C for 48 h, from which the bacterial inoculum was prepared at 10^8^ cfu ml^−1^. Periodically, the bacterium was sub-cultured on slants of the same medium and incubated (28 ± 1 °C, 48 h), before being stored at 4 °C. For the isotherms and kinetic experiments, the bacterium yield was dried and ground to the desired mesh size to get it in powder form.

### Taguchi experimental design

Methylene blue dye biosorption by *P. alcaliphila* NEWG-2 was organized during a batched fermentation process, in a functioning volume of 50 ml. Five discrete independent variables (pH, incubation time (h), initial MB level (mg/L), glucose (%), and peptone (%)) were assessed. The orthogonal Taguchi array was used to optimize MB biosorption by *P. alcaliphila* NEWG-2, at 5 levels for each factor. Accordingly, a design of 25 runs was created for the orthogonal array (L_25_). The levels of the investigated variables and the design array (L_25_) are introduced in Table 1. The experimental variation was quantified utilizing the S/N ratio, by which the biosorption quality is enhanced by minimizing the deviation of the mean square. The function type applied on the current conditions was the larger the S/N ratio the better the biosorption process, the following Eq. (1) was applied:

1 $$S/N {\text{ratio}} = - 10 \times {\text{log}}\left( {{\Sigma }\left( {1/Y^{2} } \right)/{\text{n}}} \right)$$

where n is the number of observations and *Y* is the data observed.

Upon the accomplishment of the experimental investigation, the statistically significant parameter(s) were allocated by ANOVA at probability (*P*) ≤ 0.05. Consequently, the optimum parameters’ combination of the biosorption process was determined using ANOVA, and S/N ratio. Finally, a laboratory validation trial was conducted to verify the optimum parameters’ combination that was recovered from the orthogonal array design.

### Biosorption isotherm studies

The experiments of MB biosorption isotherm studies were performed under shaking in 250 mL Erlenmeyer flasks holding 100 mL of various initial MB concentrations (10–200 ppm), and then 0.05 g of the prepared bio-sorbent material was added at the optimum pH value (8.5). Following the completion of each set of experiments, the loaded bio-sorbent material *P. alcaliphila* NEWG-2 with MB was separated from solutions by centrifugation at 10000 rpm for 20 min, and the remaining MB concentrations were determined using a UV–Vis spectrometer at 668 nm. The MB removal % from the aqueous solution was estimated by Eq. (2):

2 $$R\% = \frac{{C_{i} - C_{e} }}{{C_{i} }}*100$$

where C_i_ is the initial concentration and C_e_ is the final concentration of MB (mg/L).

Moreover, the amount of MB dye bio-sorbed (qe, mg/g) onto the surface of the prepared bio-sorbent material was estimated by Eq. (3);

3 $$q_{e} { = (}C_{0} { - }C_{e} {)}\frac{V}{M}$$

where q_e_ is biosorption capacity, C_0_ is the initial MB concentration (mg/l), C_e_ is the MB concentration at equilibrium (mg/l), M (g) is the mass of the prepared bio-sorbent materia used l, and V (L) is the volume MB solutions.

The equilibrium biosorption isotherm models are a very important step in the design of the biosorption system, as they reveal the adsorbent's capability. The biosorption isotherm plots are characterized by certain constants that definite the affinity of the bio-sorbent and its surface properties. They also, describe equilibrium relationships between bio-sorbate and bio-sorbent (the proportion of the bio-sorbed amount and the remaining amount in the solution at equilibrium)^37^. To understand the biosorption isotherms, three common equilibrium isotherm models were fitted to the obtained results. The Langmuir, Freundlich, and Temkin biosorption slopes, intercepts, and constants assessed from the isotherms plots and their correlation coefficients (R^2^) are illustrated in Table 2.

**Table 2:** Equations of the isotherm sorption models for the biosorption of MB onto the cell biomass of the bio-sorbent *P. alcaliphila* NEWG-2.

Biosorption model	Equation	Parameter
Langmuir	*C*_*e*_*/q*_*e*_ = *1/q*_*m*_*K* + *C*_*e*_*/q*_*m*_	q_e_ is the amount of MB (mg/g) bio-sorbed at equilibrium, q_m_ is the maximum capacity of the monolayer (mg/g), K is the Langmuir constant (L/mg), and C_e_ is the MB concentration at equilibrium (mg/L)
Freundlich	*Ln q*_*e*_ = *ln k*_*f*_ + *1/n*_*f*_* ln c*_*e*_	q_e_ is the amount of MB bio-sorbed at equilibrium (mg/g); C_e_ is the concentration of MB at equilibrium (mg/L); and n_f_ and K_F_ are Freundlich constants of the biosorption intensity and capacity, respectively
Temkin	*q*_*e*_ = *B ln KT ‏*+ *B ln C*_*e*_	K_T_ is the Temkin constant referring to maximum equilibrium binding energy and B is the Temkin constant of biosorption heat

The key parameters of the Langmuir isotherm model can be determined by the Langmuir separation factor, or an equilibrium parameter, R_L_, which is calculated as follows (Eq. 4):

4 $$R_{\text{L}} = \frac{1}{1 + bC_0}$$

where C_0_ is the initial MB concentration (ppm); b is Langmuir's constant; and R_L_ indicates the type of isotherm. If R_L_ values between 0 and 1 indicate a favorable biosorption, R_L_ > 1 indicates an unfavorable biosorption. Also, when R_L_ = 0 indicates irreversible biosorption, R_L_ = 1 showed a linear biosorption process.

### Biosorption kinetic studies

To investigate the kinetic studies of the MB dye molecules onto the prepared bio-sorbent material *P. alcaliphila* NEWG-2, 0.05 g of the prepared bio-sorbent material was shaken for different periods (5–120 min) with 100 mL of MB dye (10 ppm). The biosorption capacity at each time (q_t_, mg/g) was charted versus time (t, min). Table 3 shows the equations and parameters for the biosorption kinetic models that were employed.

**Table 3:** Equations of kinetic models used to describe the biosorption of MB onto the dry biomass of the bio-sorbent bacterium.

Absorption kinetic model	Equation	Parameter
Pseudo-first-order	*Ln* (*q*_*e*_ − *q*_*t*_) = *ln q*_*e*_ − *K*_1_*t*	q_t_ is the bio-sorbed dye amount at time t and q_e_ is the bio-sorbed dye amount at equilibrium (mg/g). k_1_ (min^−1^) is the constant of the first-order reaction rate
Pseudo-second-order	*t/q*_*t*_ = (*1/K*_2_*q*_*e*_^2^)	The amount of biosorbed dye at a time (t) and equilibrium, expressed in mg/g, are denoted q_t_ and q_e_, respectively. K_2_ is the second-order reaction rate equilibrium constant, expressed in g/mg min
Elovich	*q*_*t*_ = $\alpha$ + *ß ln t*	ὰ is the initial biosorption rate (mg/g min) and *ß* is the activation energy for chemisorption (g/mg)
Intra-particle diffusion	*q*_*t*_ = *k*_*id*_*t*^1/2^ + *c*	k_id_ is the intra-particle diffusion rate constant, and *c* predicts the thickness of the boundary layer

### Ascertain of the sorption process by the bio-sorbent bacterium

#### Scanning electron microscopy (SEM) inspection

The ultra-variation in the bacterial cells before and after the biosorption of MB dye was investigated by SEM (model; JEOL TEM-2100) at an accelerating voltage of 30 kV and supported with a CCD camera. The cells were coated with gold, before the examination^38^.

#### Fourier transform infrared spectroscopy (FTIR)

Prior to and following the biosorption of MB dye, the bacterial cells were examined using FTIR spectroscopy (Thermo Fisher Nicolet IS10, “USA spectrophotometer) using KBr pellets to explore the main function groups responsible for the biosorption. The spectra of bacterial cells were detected in the range of 400 to 4000 cm^−1^.

#### Software and statistical procedures

Three biological replicates were carried out. The experimental design of the Taguchi array and its statistical analysis were generated utilizing Minitab (version 21, Minitab Inc., U.S.A.) package.

## Results and discussion

Recently, the progress of new technologies has arisen in matching with overpopulation demands, due to instigating environmental equilibrium disturbance around the world. Moreover, the outbreak of unprecedented activities of industrial and urban societies has preceded the significant discharge of industrial effluent into the environment, often contaminating it with harmful organic (as dyes) and inorganic pollutants^39^.

Additionally, dyes such as methylene blue, brown 706 dye, azo dye, etc. are the most ambitious pollutant for the ecosystem, which are officious with photosynthesis as an alight obstacle into profound water as well^26^. Additionally, they threaten humanity as a result of their teratogenicity, carcinogenicity, mutagenicity, mental confusion, and jaundice^6^. However, the existence of water with good quality is essential for the long life of human beings^26^. Wherein, the eradication of such pollutants is a global concern to avoid their virulence. Currently, there are several procedures for water reclamation, however, among these procedures, the biological techniques are preferable for their compatibility with nature. Herein, one approach to these biological treatments i.e., using the bacterium of *P*. *alcaliphila* NEWG-2 as bio-sorbent for sorption of MB in two forms (dry biomass and the viable cells) has been conducted under different physicochemical conditions.

### Taguchi experimental data

Taguchi orthogonal array is usually operated to classify the best-operating settings, having a significant impact on the output parameter. The viable bacterial cells were used in the current Taguchi design. For such an aim, the process parameter is often optimized based on a larger S/N ratio is better for the output parameter. The S/N ratio determines the amount of variation in response relative to the target output under various noise conditions. In this case, the level for each tested factor that reduces the variability that occurred due to the experimental noise could be identified.

The process of MB biosorption was organized using the Taguchi approach by testing five control variables. Taguchi matrix composed of 25 runs (L_25_) for boosting the MB sorption by the bacterium. Taguchi experiments are frequently accompanied by the S/N ratio to categorize the control variables that minimize variability. Subsequently, identify the level control variables that achieve the target (maximum MB biosorption) and have no or tiny impact on the S/N ratio. Furthermore, the Taguchi approach saves time and effort by applying minimum experimental runs and, at the same time, develops satisfactory conditions^40^. The array design of the L_25_ (5^5^) and the corresponding MB biosorption of the 25 data points are introduced in Table 1 together with the predicted values by the Taguchi model. Initially, the data were analyzed, and the fitted values of MB removal were found to be very adjacent to the experimental ones, confirming the model’s prediction ability. The highest S/N ratio (39.80) was achieved by run No. L_19_, recording the maximum biosorption of MB (87.27%).

### ANOVA analysis

The data of Taguchi were subjected to ANOVA to define which of the five factors has statistically significant influences on MB biosorption (Table 4). Generally, the null hypothesis (term's coefficient is equal to zero i.e., no variation among the five factors on the MB biosorption). The significance threshold was at *P* ≤ 0.05. If a factor had a *P* value ≤ 0.05, it is considered significant, indicating a 5% connection exists when there is no real association. In this regard, each of the five factors as well as the overall model had significant effects on MB biosorption. This rule could be assured by the high *F*-value that also supports significant effects^36^.

**Table 4:** ANOVA of MB biosorption by the viable cells of *P. alcaliphila* NEWG-2 as affected by the tested factors.

Source	Freedom degree	Sum of square	Mean square	F-value	*P*-value
pH	4	1136.8	284.200	619.96	0.000
Incubation time (h)	4	135.08	33.771	73.67	0.001
Initial MB (mg/ml)	4	86.84	21.710	47.36	0.001
Glucose (%)	4	87.95	21.987	47.96	0.001
Peptone (%)	4	15.68	3.9190	8.55	0.031
Residual error	4	1.83	0.458		
Total	24	1464.18			
Determination coefficient		0.9968			
Adjusted R^2^		0.9808			

Other evaluation parameters are the determination coefficient (R^2^) and adjusted R^2^. The R^2^ and adjusted R^2^ values recorded 0.9968 and 0.9808, respectively. R^2^ value measures the change in MB biosorption response that occurred due to the changes in the factor. Normally, such parameters range between 0 to one. Higher values support the ability of the model to fit data^41^. They should not be ≤ 0.75, and if their values are ≥ 0.9, the model is highly significant^36,42^. The difference between the two parameters is that irrespective of the significance of the factor, rising the number of factors causes a constant increase in the R^2^ value. Therefore, in contrast to R^2^, the adjusted-R^2^ is a modification of R^2^ that considers the number of significant factors, consequently, changed wisely based only on the significant factor(s), leading to a real marker than R^2^ for assessing and improving the fitness of the model.

Finally, another piece of evidence of the model's accuracy was proved when, the Taguchi model was applied for the calculation of predicted MB biosorption values, which were very near to the experimental data, thus, lower errors were observed.

### Categorizing the factors and levels

The control factors and their level were classified to classify the settings of control factors that minimalize the erraticism caused by the noise factors for the best operation conditions. Therefore, the average means response for each level of each factor was estimated (Table 5), then the delta value was estimated (the subtraction of the highest and lowest average) for all control factors, and the latter were ranked based on delta values. The delta is positively correlated with the importance of the factor on MB biosorption. Hence, the results show that pH (delta 16.84, rank 1) has the supreme influence on MB biosorption, then incubation time (delta 6.84, rank 2), glucose (%), initial MB, and finally peptone (%).

**Table 5:** Mean response analysis of Taguchi data for detection of the important factors and their levels for maximization of MB biosorption by the viable cells of *P. alcaliphila* NEWG-2*.*

Level	pH	Incubation time (h)	Initial MB (mg/ml)	Glucose (%)	Peptone (%)
1	65.79	71.31	72.82	72.55	75.08
2	69.72	74.24	74.22	74.11	74.25
3	75.26	75.56	74.32	75.16	74.84
4	82.64	78.15	78.07	75.95	76.64
5	82.52	76.67	76.5	78.17	75.11
Delta	16.84	6.84	5.25	5.62	2.39
Rank	1	2	4	3	5

**Figure 1:**
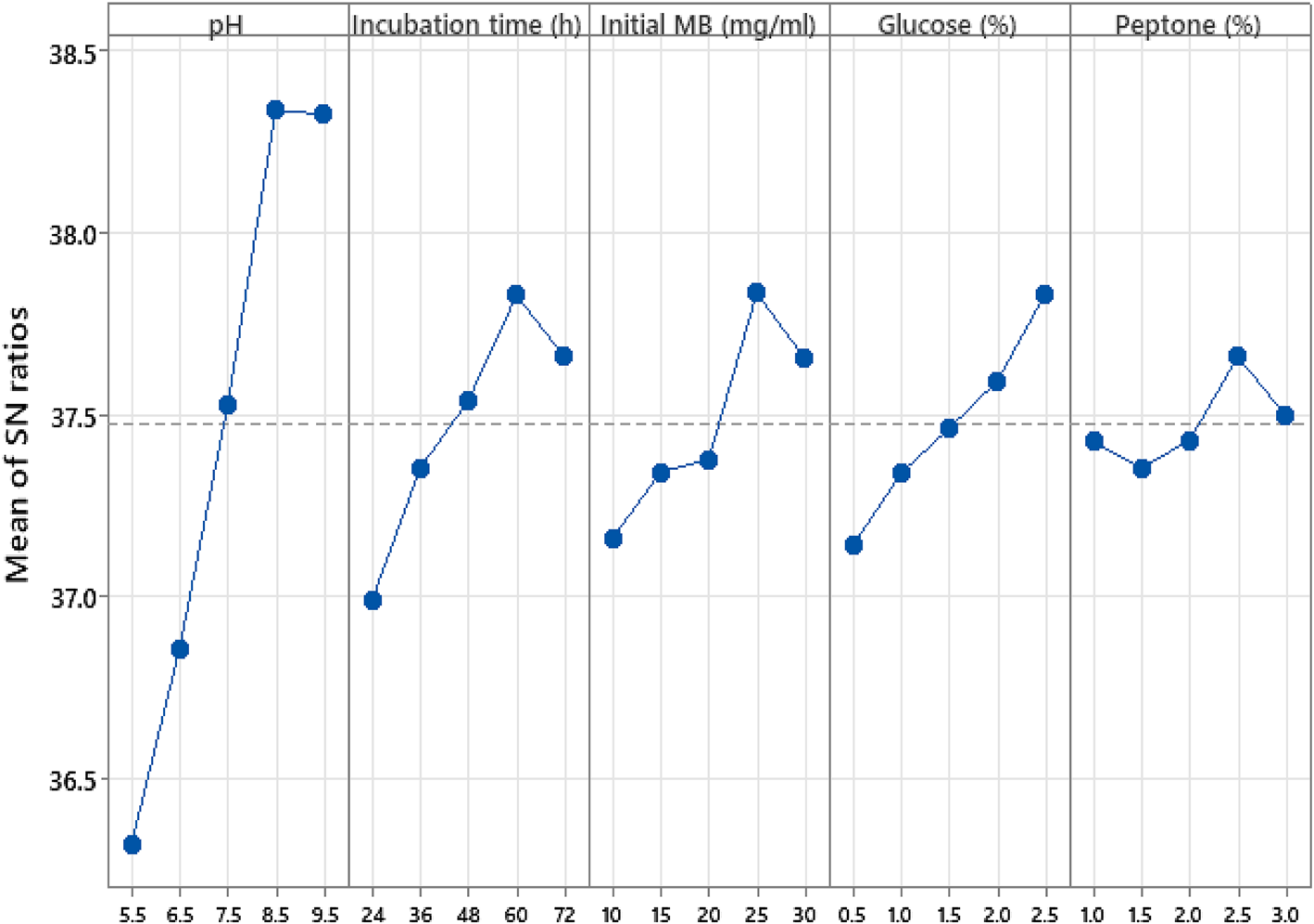
The plot of means of S/N ratios (larger is better) for MB biosorption by the viable cells of *P. alcaliphila* NEWG-2.

As depicted in Fig. 1, for the highest MB biosorption, the greatest S/N ratio for every control variable was detected at pH 8.5, incubation period 60 h, initial MB 25 mg/ml, and 25% concentration of both glucose and peptone. For each run in the Taguchi array, the mean response was calculated and the variation was based on the S/N ratio, which is altered consequently. The best levels of the current five control factors were easily determined by the robust nature of Taguchi, therefore, the variability in a process was minimized by reducing the impact of noise or uncontrollable factors during the operation. Taguchi is designed to control the noise (uncontrollable) factors during experimentation to force identify optimal control factors settings that cause the operating conditions to resist the variation caused by the noise factors. So, a high S/N ratio signifies that the control factors are at their optimal and the minimal effect of the noise factors^43^.

### Residual analysis

**Figure 2:**
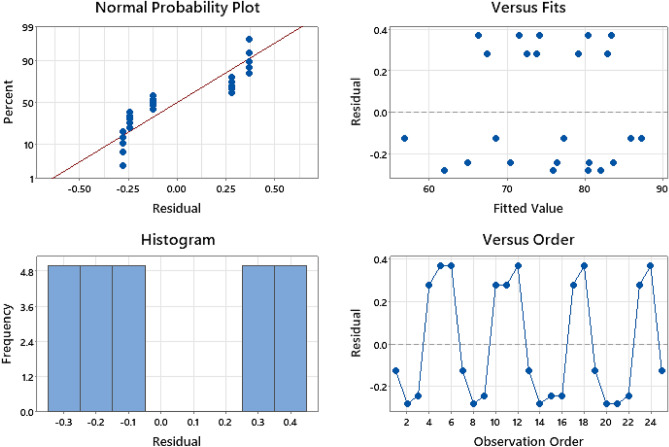
Residual plots of means for MB biosorption by the viable cells of *P. alcaliphila* NEWG-2.

Residuals are the divergence between experimental and forecasted values), and are used to evaluate whether the Taguchi model performs better during MB biosorption (Fig. 2). The plot of normal probability follows a conventional straight line without nonnormality or outliers that confirms the normal distribution of residuals. Plotting residuals against fitted values show that the points have fallen arbitrarily on both sides of the 0-axis, with no recognizable pattern, meaning that the residuals have a constant variance, and confirming their random distribution. The histogram of residuals was plotted; however, the data did not show lopsidedness or include outliers. Finally, displaying the residuals versus time order or runs signifies that the residuals fall randomly around the center line with neither a specific pattern nor trend. All previous residual analyses assume that residuals are distributed normally, which supports the appropriateness of the data for modeling MB biosorption.

### Validation of the Taguchi model

Taguchi's approach is a robust design that acts to generate foretelling information about a complex procedure with the smallest possible experimentations. Taguchi's approach successfully determined the finest mixture of five inputs that boost MB biosorption. Further, the Taguchi approach concluded the best level of the five studied factors which varied accordingly. The previously supposed null hypothesis was refused, and the alternative hypothesis proved the significance among the five control factors. Next, the fitness of the optimum levels of the five factors was experimentally authenticated using calculations from the predicted data. The biosorption level of MB was found to be 88.56%, which is very close to 87.27% recovered by run No. L19 was reported in the Taguchi array.

Compared with previous results, *Sphingomonas paucimobilis* removed 85% of MB after 5 days of retention time^44^. Another, the efficiency of *Bacillus megaterium* in removing MB reached up to 50%, by which the bacterium could tolerate the MB at the range of 29—78 mg/l, whereas, the removal efficiency of methylene blue by *Bacillus pumilis* was achieved at a concentration of 29, 58, 78 and 98 mg/l with removal efficiency being 71,74.3,67.2 and 69.9%, respectively^45^.

Concerning glucose usage in medium growth, glucose could sustain the endogenous bacteria, to biodegrade and remove the MB as an auxiliary source of carbon in addition to increasing the reduction efficiency of MB in the medium^46^.

Roy et al.^47^ investigating the optimization process of azo dye by *Pseudomonas* sp. The optimal pH, temperature, and initial dye level were pH 7, 37° C, and 50 mg/l, respectively. The highest percentage of dye removal by the biosorbent reached 79.64%. Another investigation on the decolorization of congo red by *Enterobacter cloaceae* SXCR reported that the optimum culture components (g/l) were KH_2_PO_4_ (2.2), NaCl (2.0), MgSO_4_ (0.5), glucose (2.0), peptone (3.0), and beef extract (3.0) for maximum decolorization (97%) of congo red^48^.

### Equilibrium isotherms, and kinetic processes

The dry biomass of the tested bacterium was used to study the equilibrium isotherms and kinetic processes. First, the initial concentration vis MB biosorption was determined, then the equilibrium isotherms and kinetics studies of the biosorption process were performed.

### Initial concentration vis MB biosorption

**Figure 3:**
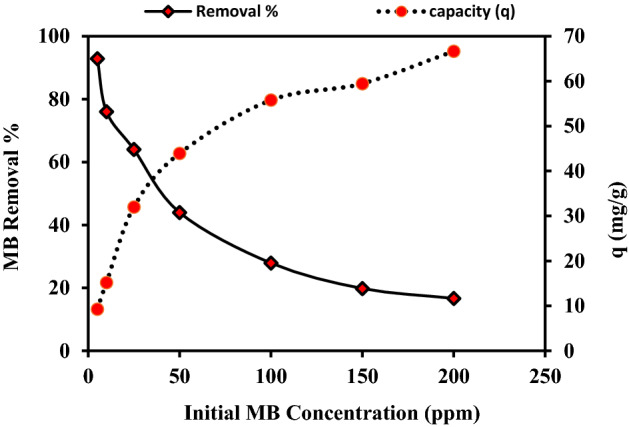
Effect of initial MB concentration on its biosorption by the biomass of *P. alcaliphila* NEWG-2.

The impact of the initial MB concentration depends upon the relationship between the binding sites on the bio-sorbent surface and the dye's initial level. The impact of the initial MB level on its removal percentage was conducted at 5, 10, 25, 50, 100, 150, and 200 ppm using 0.05 g of the prepared bio-sorbent material for 60 min (Fig. 3). As the MB concentration rise from 5–200 ppm, the percentage of MB dye removal decreased from 92.857 to 16.667%, demonstrating that the dye removal percentage is inversely correlated to its initial concentration. Contrarily, the biosorption capacity (q) increases as the initial solution concentration increases, rising from 9.286 to 66.667 mg/g as the initial MB concentration increased from 5–200 ppm. This could be because, at lower dye concentrations, there are numerous active sites on the surfaces of the prepared bio-sorbent materials ready for the biosorption of the MB molecules. While, with the increment in dye concentration, these adsorption active sites became occupied and reached saturation rapidly, which increase the repulsion force between dye molecules and subsequently decreased its removal percentage^49^. In addition, this behavior could be explained by an increase in driving force brought on by the concentration gradient created by raising the initial dye concentration, which overcomes the MB molecules' resistance to mass transfer between the aqueous and solid phases^50^. The same trend was also observed by Fakhry et al.^51^ and El-Aassar et al.^52^.

Bhattacharyya et al.^53^ reported that the adsorbed amount of MB increased from 11.63 to 30.66 mg/g as the concentration of MB increased from 25 to 70 mg/L. Moreover, Kilany^54^ mentioned that the MB removal percentage increased with the increase in its initial concentration, reaching its peak at 5 mg/l (61.3%); any additional increase in the initial concentration had no significant increase in the dye removal percentage. Furthermore, Yi et al.^55^ demonstrated that the peak adsorption amount of ANFs/BC-1 was 75.06 mg/g at an initial concentration of 200 mg/l.

### Biosorption isotherms

**Figure 4:**
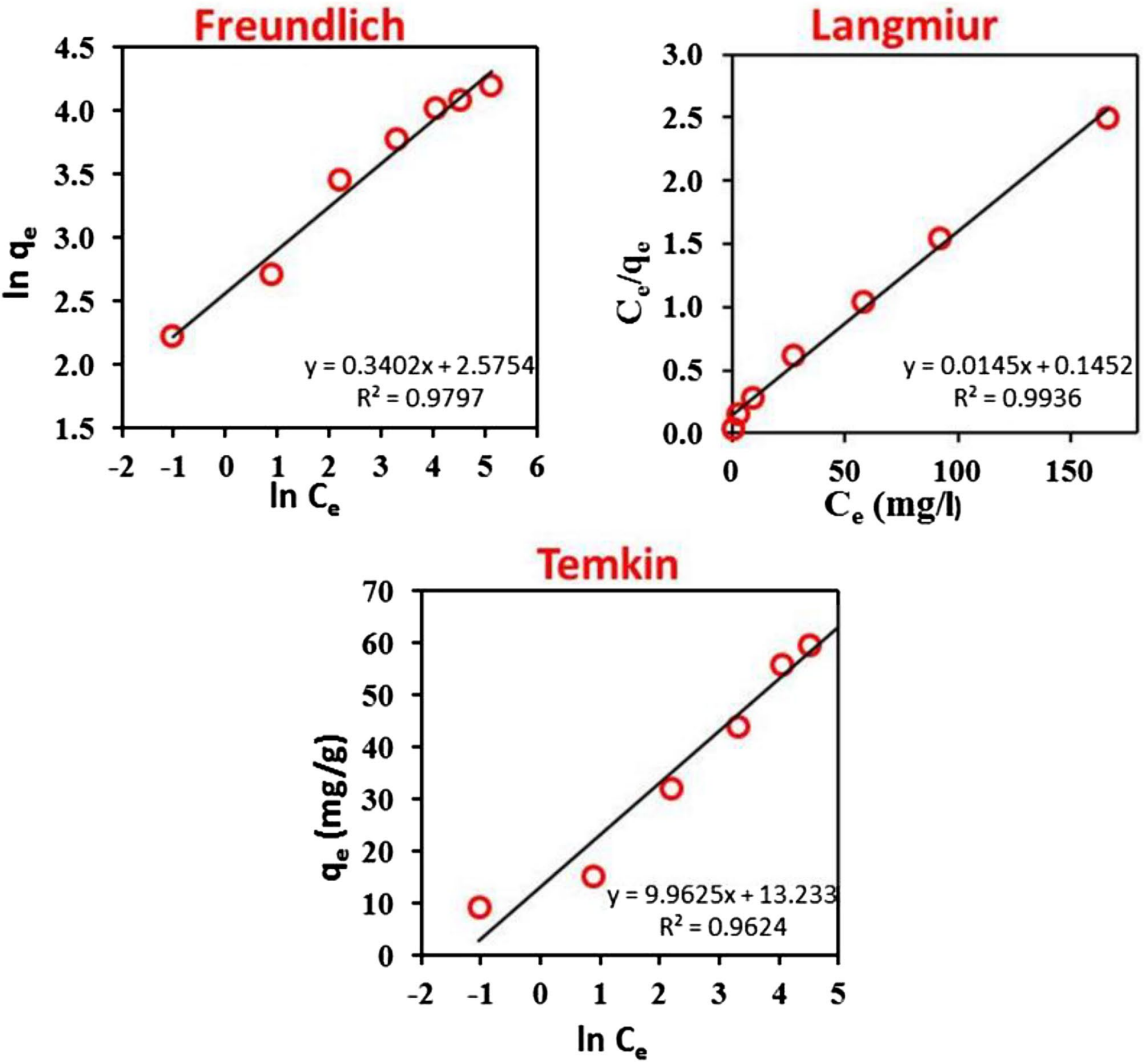
Models for equilibrium isotherms for the biosorption of MB onto the biomass of *P. alcaliphila* NEWG-2 bio-sorbent material include Langmuir, Freundlich, and Temkin.

The obtained data from investigating the effect of different MB dye initial concentrations on the biosorption capacity of the prepared bio-sorbent were fitted to the chosen isotherm models. All the calculated parameters from different isotherm model plots are illustrated in Fig. 4 and Table 6.

**Table 6:** Langmuir, Freundlich, and Temkin parameters for the biosorption of MB onto the biomass of *P. alcaliphila* NEWG-2 bio-sorbent.

Isotherm	Parameter	Value
Langmuir	q_m_ (mg g^−1^) calculated	68.827
	K_L_ (L mg^−1^)	0.100
	R^2^	0.993
Freundlich	K_F_ (mg1^−1^/n L1/n g^−1^)	376.150
	Nf	2.940
	R^2^	0.979
Temkin	B (L mg^−1^)	9.962
	K_T_ (KJ mol^−1^)	3.775
	R^2^	0.962

The Langmuir isotherm model assumes monolayer coverage of MB molecules over a homogeneous biomass surface, identical biosorption active sites, and uniform biosorption energies^56,57^. The Langmuir model has a high R^2^ (0.997), with a maximum biosorption capacity of MB dye molecules onto the prepared bio-sorbent of 68.827 mg/g. These findings suggest that the biosorption of MB dye molecules onto the prepared bio-sorbent takes place in monolayer coverage, and at specific sites over the prepared bio-sorbent material surface, so, there is no additional binding process at the active sites once they are occupied^58^. In, the Langmuir constant R_L_ value (R_L_ = 0.500) indicated the favorability of MB biosorption onto the prepared bio-sorbed material. The Freundlich isotherm does not show data regarding the monolayer biosorption capacity of the bio-sorbent matrix, in difference to the Langmuir model. The heterogeneous site energies and unrestricted degrees of biosorption are assumptions made by the Freundlich isotherm model. The main characteristic of heterogeneous surfaces, an exponential distribution of active sites and biosorption energies, is also assumed^59,60^. The correlation coefficient value obtained from the Freundlich isotherm plot (R^2^ = 0.979) was lower than the R^2^ value of the Langmuir isotherm, revealing that the Freundlich model does not accurately describe the behavior of saturation of the prepared bio-sorbent, with constant energy of biosorption, and there is no transmigration of MB molecules to the inner layers.

The Temkin isotherm model includes the interactions of biosorbent-biosorbate. This model supposes a heterogenous biosorption of bio-sorbate on the surface of the bio-sorbent. Additionally, rather than declining logarithmically, the heat of biosorption is falling linearly instead^57^. The values of K_T_ and b can be acquired from the slope and intercept of the Temkin equation (Table 6).

The value of the correlation coefficient of the Temkin plot (R^2^ = 0.96) is significantly less than the isotherms of Langmuir and Freundlich which reveal unequally distributed binding energy developing as a result of the dye molecules' interaction^50^. The current study revealed an obvious improvement in the Langmuir biosorption capacity compared with several former studies (Table 7).

**Table 7:** The current study of Langmuir biosorption capability compared to that of previous studies.

Biosorbent	Biosorption capacity (mg/g)	References
Sugarcane bagasse	9.41	^61^
*Aspergillus carbonarius* (AC)	21.88	^61^
*Penicillium glabrum* (PG) fungi	16.67	
*Terminalia catappa* (TC) shells	88.62	^62^
Lemna minor (L. minor)	1.07	^63^
*Cyanthilium cinereum* (L.) weeds	56.18	^64^
*P*. *alcaliphila* NEWG-2	68.83	Current study

### Contact time vis MB biosorption

**Figure 5:**
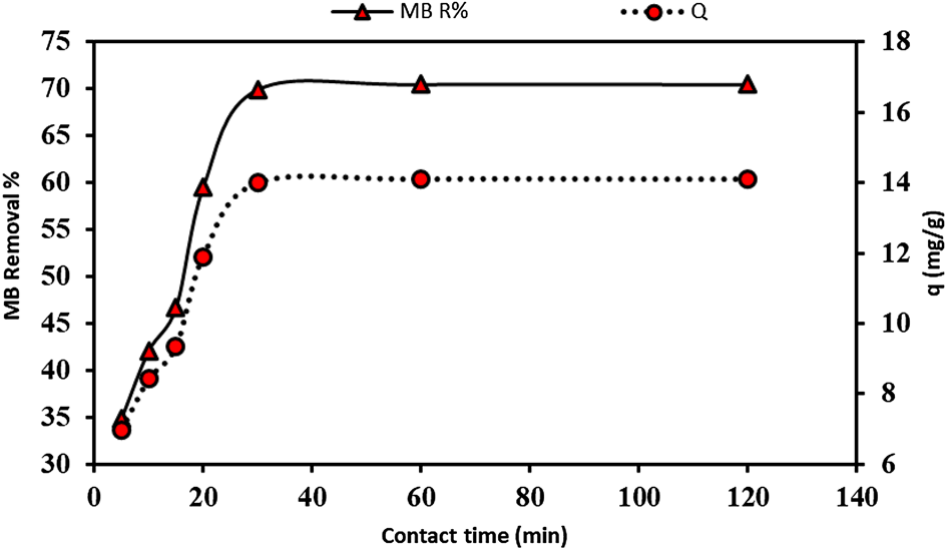
Contact time vis MB biosorption by the biomass of *P. alcaliphila* NEWG-2 bio-sorbent.

The impact of contact time on MB biosorption onto the prepared bio-sorbent was examined over 120-min time intervals, as illustrated in Fig. 5. MB R% rose as contact time increased until equilibrium was established at 60 min for an initial dye concentration of 10 mg/l. After the equilibrium time, the removal percentages remained almost constant with longer contact times.

The MB removal % onto the prepared bio-sorbent increases from 34.853 to 70.509% when the contact time increases from 5 to 120 min, that might be because there are several empty surface-active sites available for biosorption in the first stage, additionally, the residual empty sites are difficult to access after time has passed due to the repulsive forces between the MB molecules and the bio-sorbent. Moreover, it can be predicted that at the beginning of contact time between the prepared bio-sorbent materials and MB dye molecules, the biosorption takes place on the prepared bio-sorbent materials surface, hence, the biosorption rate is rapid. Once the surface is saturated, MB diffuses into the material's interior, where adsorption continues, and subsequently lowers the adsorption rate^6,65^. Eltarahony et al.^18^ reported an equilibrium time of 2 h. Also, Ugraskan et al.^66^ mentioned that the ideal contact time was 90 min for the adsorption of MB onto porous boron carbide. While Ugraskan et al.^66^ reported that the optimum contact time of MB onto Judas tree (*Cercis siliquastrum*) seeds was determined to be 60 min, which agreed with our results.

### Biosorption kinetic studies

The biosorption process of bio-sorbate onto bio-sorbent bacterium under stirring conditions is typically controlled by several transport stages; external diffusion, internal diffusion, and biosorption on the pore surface, or a mixture of more than one step^49,67^. The biosorption of MB onto the prepared material was studied to determine the rate-controlling steps and mechanism.

**Figure 6:**
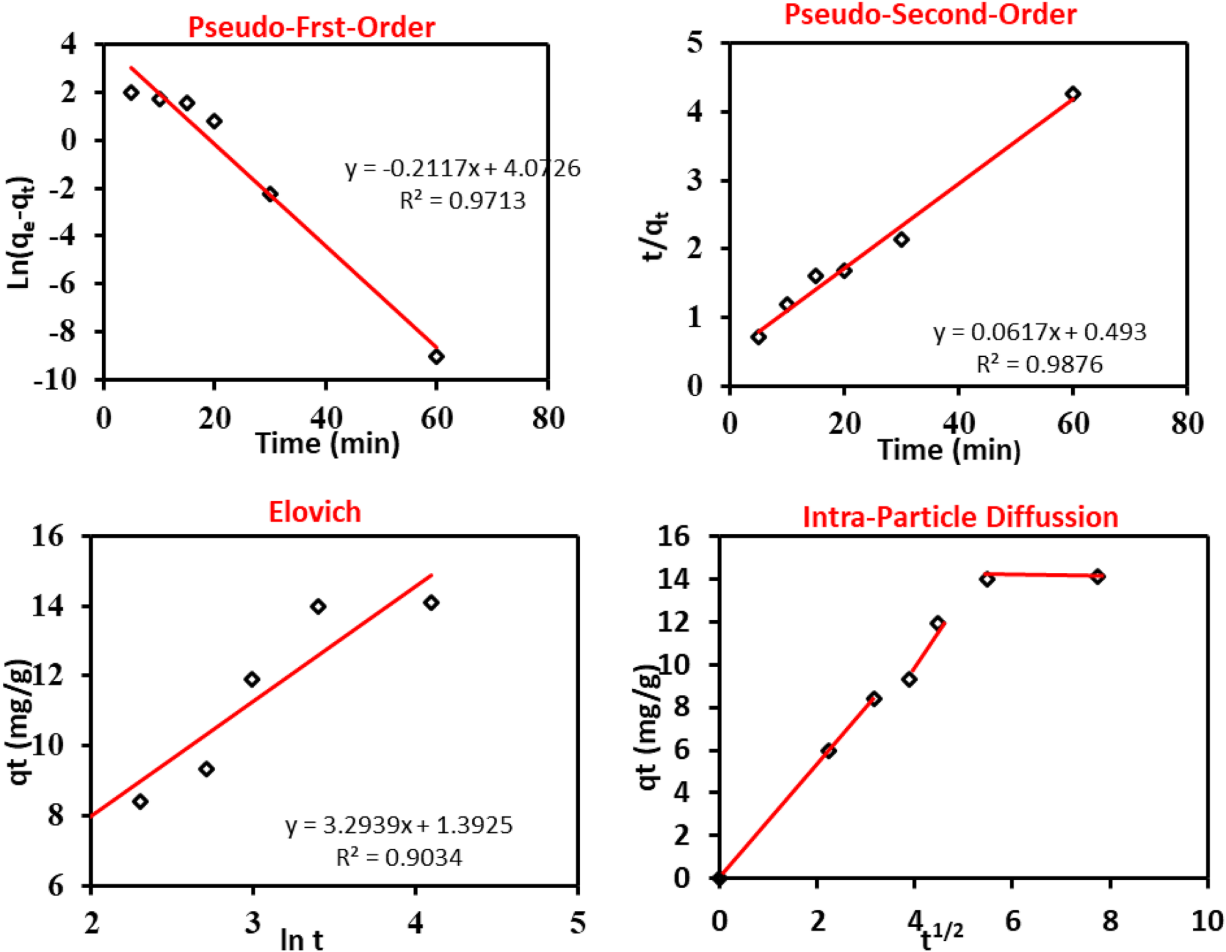
The different kinetic models that were applied on MB biosorption onto the prepared *P. alcaliphila* NEWG-2 bio-sorbent.

Investigating the effects of biosorption time of the MB dye biosorption onto the used bio-sorbent material was performed; the experimental results were modeled using pseudo-first-order (PFO), pseudo-second-order (PSO), Elovich, and intra-particle diffusion models. Figure 6 displays a linearization form plot for a PFO model. The PFO constant and equilibrium adsorption were determined from the plot's slopes and intercepts (Table 8).

**Table 8:** Parameters for the biosorption of MB dye onto the used *P. alcaliphila* NEWG-2 bio-sorbent material obtained from different kinetic models.

Kinetic model	Parameter	Value
Pseudo-first-order	q_e_ (mg/g) Calculated	58.711
	q_e_ (mg/g) Experimental	14.102
	k_1_ (min^−1^)	− 0.004
	R^2^	0.971
Pseudo-second-order	q_e_ (mg/g) Calculated	16.215
	q_e_ (mg/g) Experimental	14.102
	k_2_ (g/mg min)	0.008
	R^2^	0.989
Elovich	ß (g/mg)	3.29
	ὰ (mg/g min)	1.392
	R^2^	0.903
Intra-particle diffusion	ki_d_	1.53
	I	3.745
	R^2^	0.830

The correlation coefficient describes how closely the experimental results were consistent with model predictions. A high R^2^ value means that the model accurately describes the MB biosorption kinetics. In this model, the experimental value of biosorption capacity deviated from the calculated values, although, the model plot has a high correlation coefficient (PFO R^2^ = 0.971).

The identified deviation from the obtained data has been explained by the sharp fall in a gradient of concentration after the initial fast biosorption of MB molecules in the early stages of the biosorption, due to the presence of a significant amount of unoccupied sites for biosorption. During this time, there might be a shifting between pore diffusion control, and mass transfer diffusion control^68^, which means that the PFO doesn’t fit to describe the biosorption kinetic process of MB dye onto the used bio-sorbent material.

For the applicability of the PSO to the obtained data, the correlation coefficient of the PSO model values attained from the linear plot of t/q_t_ versus t was (R^2^ = 0.99) for the biosorption kinetic process of MB dye onto the used bio-sorbent material, which means that the chemisorption is the rate-limiting step in this process, not the boundary layer resistance with sharing electrons between bio-sorbate and bio-sorbent^69,70^. The theoretical biosorption capacity (q_e, cal_ = 14.102 mg/g) value was compared with their experimental values (q_e, exp_ = 16.21 mg/g), which were so close. Figure 6 and Table 8 show the plot of the experimental data of MB amount bio-sorbed per unit mass of the prepared bio-sorbent against time along with the model values for the PFO and PSO models. The PFO model correlation coefficient is less than the PSO coefficient, so, the PSO kinetic model produced a good correlation. for the biosorption of MB dye onto the used bio-sorbent material. The proximity of the equilibrium capacity of the PSO model to the experimental equilibrium capacity reveals the fitness of the PSO model to designate the kinetics of MB biosorption in the investigated contact time range.

Elovich equation model has been widely used in studying the kinetics of adsorption; it depicts the chemical reaction (adsorption) mechanism in nature, as well as, it is suitable for heterogeneous bio-sorbing surfaces systems, and they offer various activation energies based on a second-order reaction mechanism for reaction with a heterogeneous process^71^.

The high correlation coefficient (R^2^ = 0.903) values reveal that the Elovich equation matches the experimental data well, assuming that a chemisorption process (chemical biosorption) between the MB molecules and the used bio-sorbent material may contain valence forces via sharing electrons between them. Thus, both the PSO and Elovich equation fitted well with the experimental data which points to a chemisorption interaction type between MB dye and the used *P. alcaliphila* NEWG-2 bio-sorbent material. The same trend of chemical reaction, as the rate-controlling step, was previously reported^72^.

The intraparticle diffusion design model by Weber and Morris has been extensively used to study biosorption kinetics. According to this model, the rate-limiting step is the intraparticle diffusion if a plot of the solute sorbed (q_t_) against the square root of the contact time (t^1/2^) results in a straight line passing through the origin^73^. The multi-linearity in the intraparticle diffusion plots indicates that there are three main stages in the biosorption process. The first, noticeable stage is caused by MB molecules' diffusion through the aqueous phase to the external surface of the prepared bio-sorbent material or diffusing across the boundary layer. The gradual biosorption is described in the second stage, where the rate-limiting step is intraparticle diffusion. The 3rd stage is referred to as the last equilibrium step, where the intraparticle diffusion starts to slow down because there isn't much MB left in the solution. The obtained three stages indicate that the biosorption process takes place by surface biosorption and intraparticle diffusion (meso- and micropores). The same trend of the multi-linearity plot of the intraparticle diffusion has also been reported and used the giant duckweed in the biosorption of MB. El-Khaiary^74^ also utilized the biosorption of methyl violet and MB by mansonia wood sawdust^75^. The intraparticle diffusion parameters of the models were estimated (Table 8).

### The bacterial surface topology investigation by SEM analysis

**Figure 7:**
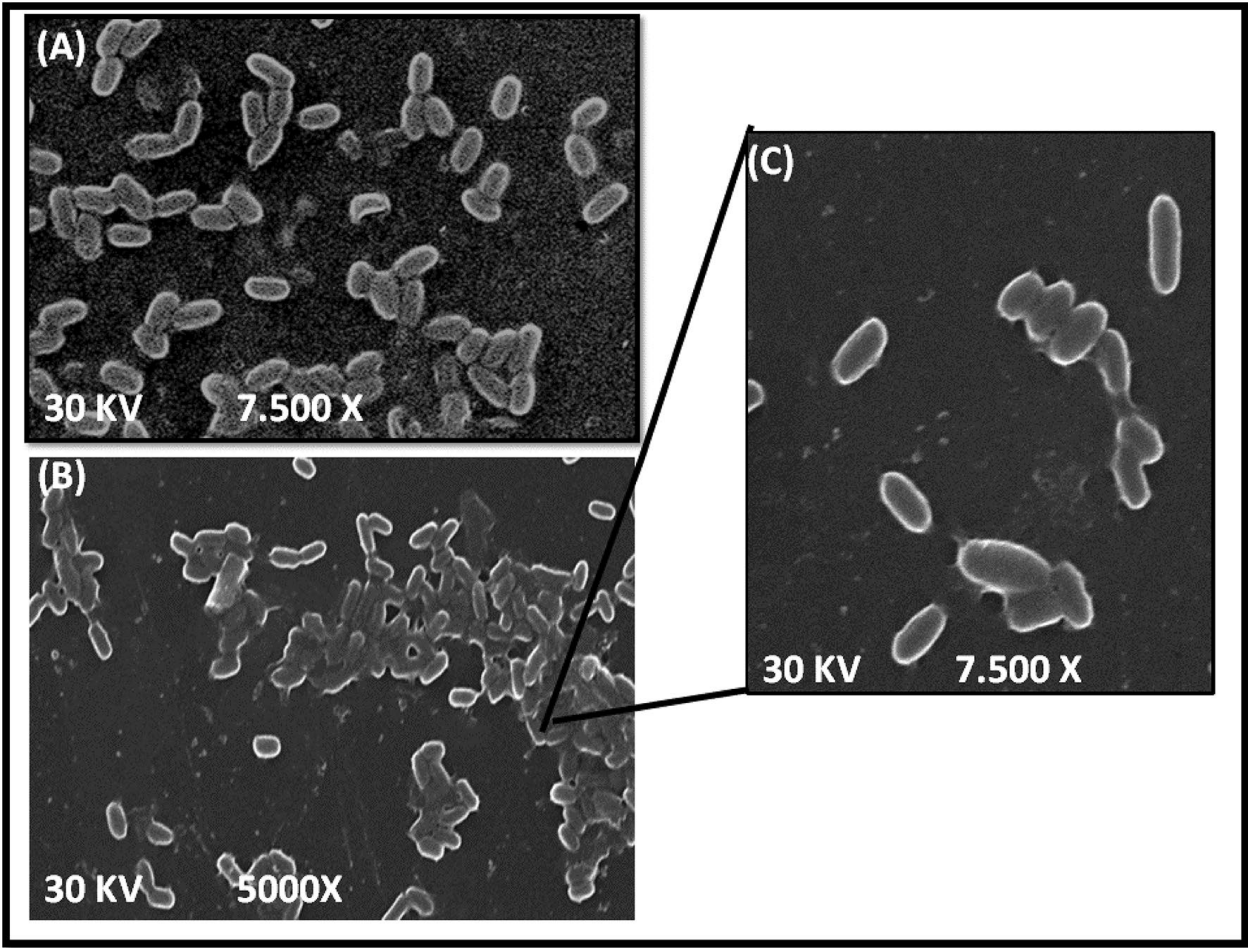
SEM image of *P. alcaliphila* NEWG-2 without MB (**A**), and bacterial cells treated with MB (**B**,**C**) at different magnifications.

The SEM is a helpful tool for displaying the morphological characteristics, and surface topology of isolated organisms^76^. A specimen of a bacterial cell before and after interacting with MB molecules was investigated by the SEM technique (Fig. 7) at different magnifications. SEM images showed that the cells of *P*. *alcaliphila* NEWG-2 showed a smooth, and rod-shaped appearance with well-defined edges as single-cell distributions without clustering before their biosorption of MB molecules (Fig. 7A), while after interacting with methylene blue, there was a noticeable aggregation of bacterial cells which can be attributed to the production of a sticky matrix likely to be an exogenous polysaccharide induced by different stress conditions. *Pseudomonas* bacteria have been shown in several studies to produce exopolysaccharides as an adaptive mechanism under different stress conditions such as cold, pH, or in this case the treatment with MB^77–79^. both of the bacterial cells' surfaces before and after the biosorption process have the same shape, dimensions, and texture, which might be because these cells form a type of polysaccharides as a defense mechanism to tolerate MB^80^.

### FTIR spectra analysis

**Figure 8:**
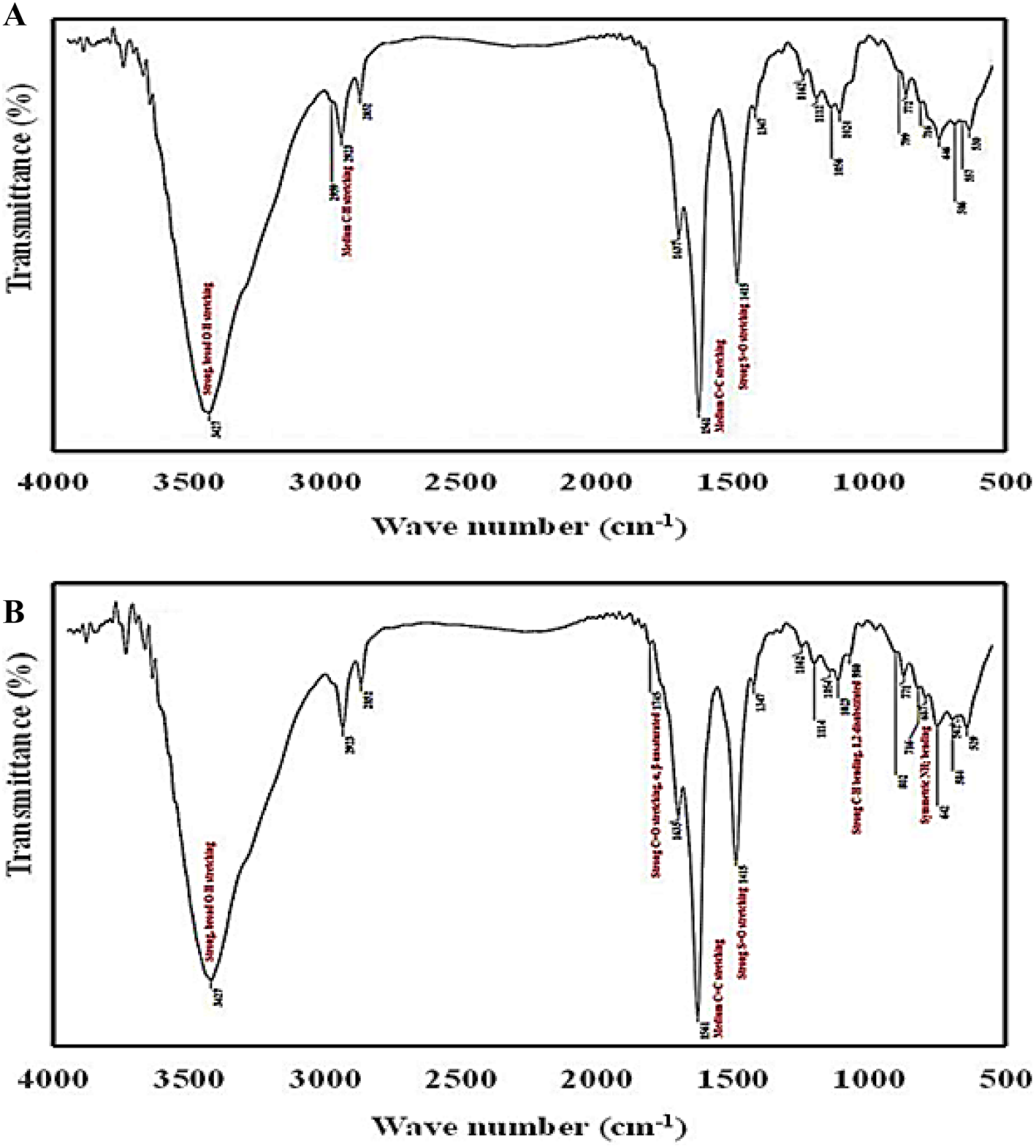
*P. alcaliphila* NEWG-2 spectral charts of the FTIR analysis of control (**A**), and treated with methylene blue dye (**B**).

The infrared (IR) spectroscopic analyses before and after MB-biosorption dye were investigated, to characterize the frequencies and the interpreted functional groups (Fig. 8). The IR spectra of the control and, MB-treated bacterium, were investigated. When in contact with MB dye, the features of the bacterial surface morphologically changed. In general, the IR spectra showed changes in the functional groups' frequency before and after MB biosorption because of forming new bonds between the various groups, leading to the formation of other new bonds, or the diffraction of some other group frequencies. The data of IR spectra showed an absorptance band at ν = 3423–3445 cm^−1^ which is due to the strong or wide stretching vibration of hydroxy (O–H) groups.

Another new absorption strong stretching band was recorded for MB-treated *P. alcaliphila* NEWG-2 at ν = 1745 cm^−1^ due to the formation of C = O (α, β-unsaturated ester) group. The frequency values within the spectral IR range of ν = 1637–1024 cm^−1^ showed the presence of eight groups in both samples without variation in the absorption bands, just slightly shifted values. However, the stretching of absorption bands; strong C = O, amide, medium C = C, strong S = O, medium C-H bending, strong C-O, strong C-O bands (secondary alcohol), strong C-O stretching (primary alcohol), and strong stretching S = O or Si–O-Si groups were recognized at ν = 1634–1638, 1561–1562, 1414–1416, 1347–1349, 1160–1162, 1109–1114, 1052–1059, and 1023–1024 cm^−1^, in sequence. The control sample did not demonstrate absorption bands at ν = 935–980 cm^−1^, whereas the MB-treated sample displayed absorption bands due to the strong C-H bending groups. The bands at ν = 772–880 cm^−1^ are attributed to the strong C-H bending (1,2,3-trisubstituted) in both samples but with slight shifting in the control sample. In the range of ν = 683–687 cm^−1^, no band was noticed in the control sample, whereas in the MB-treated one, the absorption bands of symmetric amino (NH_2_) bending appeared. The values of frequencies at ν = 582–586 and 624–646 cm^−1^ are attributed to the stretching vibration bands of C-halogen groups or aromatic rings. Additionally, the spectra of the non-treated bacterium revealed an absorption band at ν = 530 cm^−1^ owing to a strong stretching vibration of halogen linked to the ligand. These results are conceding with that obtained during the biosorption of brown706 dye by *Pseudomonas aeruginosa*^26^. Another study discussed the FTIR spectra analysis of active sites of *Pseudomonas* sp. during the biosorption of azo dye^47^.

## Conclusion

Summing up, the Taguchi array has been performed to maximize the biosorption of MB by *P. alcaliphila* NEWG-2. This exclusive study was also supported by biosorption isotherms and kinetic studies. Taguchi approved its efficacy for modeling the bacterial biosorption with a maximum biosorption of 87.14% at pH 8, after 60 h under the optimized condition which is very promising. Equilibrium isotherms and kinetic processes were validated especially with the Langmuir model (q_max_ = 68.827 mg/g). The equilibrium time of MB biosorption was relatively short at 60 min. The biosorption kinetic profile obeyed PSO and Elovich models. SEM analysis of bacterium cells showed conspicuous variation in the bacterial cell surfaces. FTIR also showed various functional groups on the bacterial cell wall that participated in the biosorption of MB. *P. alcaliphila* NEWG-2 could be recommended as a talented bio-sorbent for the decolorization and remedy of an industrial effluent containing MB.

## Data Availability

This published article contains all the data that was generated or analyzed during the study.
